# Dietary patterns, nutritional status, and mortality risks among the elderly

**DOI:** 10.3389/fnut.2022.963060

**Published:** 2022-12-09

**Authors:** Zhen Liu, Di Xu, Wen-xiu Xu, Yin-jiao Fei, Dan-dan Wang, Fei Deng, Jin-hai Tang

**Affiliations:** ^1^Department of General Surgery, The First Affiliated Hospital of Nanjing Medical University, Nanjing, China; ^2^Department of General Surgery, Pukou Branch of Jiangsu People’s Hospital, Nanjing, China

**Keywords:** diet, k-means clustering, mortality, nutritional status, dietary patterns

## Abstract

**Introduction:**

While most epidemiological studies have focused on the effects of individual dietary patterns and nutritional status on health, the relationships between the combinations of these factors and patient prognosis requires further investigation.

**Objective:**

This study explored mortality risk in individuals with different combinations of dietary patterns or nutritional status.

**Methods:**

Unsupervised K-means clustering was used to classify populations. The analyses included Cox proportional risk and competing risk models.

**Results:**

After considering a complex sampling design, the results showed that among 12,724 participants aged >60 years, 6.99% died from cancer and 10.47% from cardiovascular and cerebrovascular disease (CCVD). After correcting for participant baseline information and chronic conditions, the geriatric nutritional risk index and healthy eating index (HEI) were negatively associated with the risk of all-cause and cause-specific mortality. The opposite was true for the dietary inflammatory index (DII). After sorting the population three clusters based on study scores showed higher risks of all-cause mortality and cancer-related death in Cluster 2 and 3.

**Discussion:**

These results suggest that different nutritional status and dietary patterns are associated with the risk of all-cause mortality and death from cancer and CCVD in people aged >60 years in the United States. Dietary patterns with high HEI and low DII were beneficial to health, whereas nutritional status needs to be maintained at a level that is not too low.

## Introduction

Typical dietary patterns are practical nutritional tools that reflect regular dietary habits. A nutritional gap in the diet refers to specific nutrient deficits that may lead to deficiencies and poor health. Dietary and nutritional factors may contribute to or mitigate disease development and significantly influence its outcome (1). The indicators commonly used to measure dietary patterns and nutritional status include the dietary inflammatory index (DII), Healthy Eating Index (HEI), and Geriatric Nutritional Risk Index (GNRI).

Assessing the nutritional status of individuals is complex. The GNRI is widely used to measure nutritional status in older people, as it is derived from serum albumin levels and body mass indexes (2, 3). A scoring algorithm was developed for the DII to estimate an individual’s dietary inflammatory potential (4). The National Cancer Institute developed the HEI to evaluate dietary quality in the US (5). While epidemiological studies have explored the relationship between the GNRI (nutritional indicator) and DII and HEI (dietary indicators) and all-cause, cardiovascular and cerebrovascular disease (CCVD), and cancer mortality (6–11), these conclusions remain inadequate. In addition, most studies assess one dimension of nutrition or dietary habits, with few studies focusing on the health effects of the multiple dimensions of food and nutritional habits.

The impact of different dietary patterns and nutritional status combinations on health warrants exploration. Classifying individuals according to different combinations can provide information on their dietary habits, nutritional status, and health in a broader dimension. This study examined this effect using information from the National Health and Nutrition Examination Survey (NHANES) database from 1999 to 2018. We explored the relationship of dietary patterns and nutritional status with all-cause and cause-specific mortality by classifying populations using k-means clustering methods.

## Materials and methods

### Study design

**FIGURE 1:**
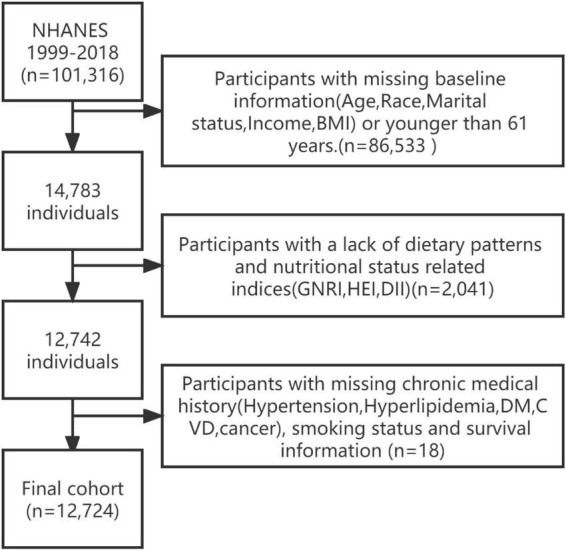
A flow chart for selecting cohort study participants.

This prospective study included adults aged >60 years from 1999 to 2018. The NHANES is a cross-sectional, ongoing study of the US non-institutionalized population and includes data from household interviews, examinations, and post-examinations. The samples were selected using a complex multistage process. The participants’ publicly available mortality data were obtained on December 31, 2019 (12). According to the International Classification of Diseases, the primary outcomes were all-cause mortality, as well as cancer (C00-C97), cardiac (I00-I09, I11, I13, I20-I51), cerebral (I60-I69), and vascular disease (CCVD). The participant selection and exclusion flow chart was shown in Figure 1. The NCHS research ethics review board approved the NHANES protocol.

### Nutritional status and dietary patterns

The GNRI is calculated using the following formula:


$$\mathrm{GNRI}=\left[1.489\times \mathrm{Alb}\,\left(g/L\right)\right]+\left\{41.7^{*}\,\left[\mathrm{weight}\,\left(\mathrm{kg}\right)/\mathrm{IBW}\,\left(\mathrm{kg}\right)\right]\right\}.$$

Where Alb is serum albumin and the ideal body weight (IBW) indicates a body mass index (BMI) of 22 kg/m^2^ (2).

The DII is an indicator of dietary inflammation that is used to assess the anti-and pro-inflammatory properties of the diet of different individuals. Specific descriptions are available elsewhere (4). Based on a literature review, the DII was used to report the effects of 45 different dietary or nutritional intakes on inflammatory markers [interleukin (IL)-1 β, IL-4, IL-6, IL-10, C-reactive protein, and tumor necrosis factor-alpha (TNF-α)]. The intake of each food component was subtracted from its standard evaluated intake and divided by its standard deviation. The resulting value was converted to an intermediate percentage and multiplied by the overall inflammatory effect score for the food component. The DII score is the sum of the food scores. A positive DII value indicates a pro-inflammatory diet, whereas the opposite is true for an anti-inflammatory diet. In our study, the food components included: carbohydrates, proteins, total fat, alcohol, fiber, cholesterol, saturated fat, monounsaturated fatty acids, polyunsaturated fatty acids, n-3 fatty acids, n-6 fatty acids, niacin, vitamins (A, B1, B2, B6, B12, C, and E), iron, magnesium, zinc, selenium, caffeine, and energy.

Diet quality was assessed using the 2015 version of the HEI (5), which included 13 ingredients (whole protein foods, total vegetables, whole fruits, whole grains, greens and beans, dairy, seafood, plant proteins, fatty acids, sodium, added sugars, refined grains, and saturated fats). The HEI is calculated based on density (amount of food components/1000 kcal). The higher the score, the higher the diet quality.

### Covariates

The covariates in this study population included age, sex, race, household income, BMI, marital status, and smoking status. The baseline information of these participants was obtained from the responses to the NHANES’ demographics or questionnaires. Hyperlipidemia was defined as (1) triglyceride level ≥ 150 mg/dl; (2) total cholesterol level ≥ 200 mg/dl, low-density lipoprotein level ≥ 130 mg/dl, high-density lipoprotein level < 40 mg/dl (male) and <50 mg/dl (female), or (3) treatment with lipid-lowering drugs. Hypertension was defined as (1) self-reported use of hypertensive medication, (2) previously reported hypertension (from a questionnaire), or (3) current blood pressure measurement (mean systolic blood pressure ≥ 140 mmHg or mean diastolic blood pressure ≥ 90 mmHg). Diabetes mellitus (DM) was defined as (1) self-reported or told by a physician to have diabetes; (2) glycated hemoglobin ≥ 6.5%; (3) fasting glucose ≥ 7.1 mmol/L, DM; ≥6.11 and <7.0, impaired fasting glycemia; (4) random glucose ≥ 11.1 mmol/L; (5) 2 h oral glucose tolerance test ≥ 11.1 mmol/L, DM; ≥7.7 and <11.1 mmol/L, impaired glucose tolerance; (6) self-reported use of diabetes medication. Smoking was defined as (1) having smoked less than 100 cigarettes, no smoke; (2) having smoked more than 100 cigarettes and not currently smoking, former smoke; and (3) having smoked more than 100 cigarettes and currently smoking, now smoke. NHANES provides more details on the above covariates (websites in the Supplementary material). Publicly available participant mortality data were used to determine the causes of death of the patients who died.

### Statistical analysis

Following the guidelines for using the NHANES database, sampling weights were considered in the analysis of the relationship between the variables and all-cause mortality. Weighted analyses were conducted to better reflect the overall picture. Categorical and continuous variables are expressed as percentages (%) and co-medians (interquartile range), respectively. Nutritional and dietary pattern scores were log-transformed and standardized for analysis. Differences in categorical and continuous variables were tested using Rao–Scott chi-square and Wilcoxon rank-sum tests. We used k-means clustering to identify groups of individuals with similar characteristics. K-means, a popular unsupervised algorithm, can be used to distinguish between groups. It is widely used in the medical field (13, 14). K-means groups patients by identifying the centroids of different groups. It was first proposed in 1967 (15). The algorithm divides the participants into clusters where the number of collections k is chosen autonomously. We used the “factoextra” package (16), clustering effects, and clinical significance to find the optimal number of clusters. The final result was three cluster centers, with the population belonging to each group labeled. Rao–Scott chi-square tests were used to compare the baseline characteristics of the participants in different clusters. Bar charts were used to describe the dietary structure or nutritional attributes of the different populations.

We used the Cox proportional risk model to determine the associations of all-cause mortality with variables and clusters and calculated the hazard ratios (HRs) and corresponding 95% confidence intervals (95% CIs). We performed competing risk analyses to compare different variables and clusters of cancer-specific or CCVD deaths and obtained HRs (17). The three models in the study were as follows: model one, which included only the study variables and did not adjust for other factors; model two, which adjusted for age, sex, race, marital status, and household income; and model three, which additionally adjusted for smoking status, hypertension, hyperlipidemia, and diabetes. Ten participants were not included in model three owing to a lack of data on smoking status. All analyses were performed using R version 4.1.0. All statistical analyses were two-sided. Statistical significance was defined as *p* < 0.05.

## Results

### Baseline characteristics of participants

After excluding participants with missing study variables and covariates, this study included 12,724 participants aged >60 years. After weighting, 6.99% died of cancer and 10.47% died of cardiovascular disease over a median follow-up time of 7.1 years. The weighted baseline characteristics of the participants are shown in Tables 1, 2. The unweighted baseline characteristics are shown in Supplementary Table 1.

**TABLE 1:** Baseline demographic characteristics of all participants in this study.

Characteristics	Total	Female	Male	*P*-value*
Age (years)	70.00 (65.00, 76.00)	70.00 (65.00, 77.00)	69.00 (65.00, 76.00)	< 0.001
BMI (kg/m2)	28.07 (24.83, 32.09)	28.01 (24.38, 32.50)	28.10 (25.35, 31.70)	0.23
GNRI	115.23 (108.40, 122.94)	114.98 (107.96, 123.41)	115.47 (108.90, 122.36)	0.76
DII	1.30 (-0.32, 2.56)	1.66 (0.13, 2.78)	0.77 (-0.72, 2.17)	< 0.001
HEI	56.31 (47.17, 66.17)	57.42 (48.40, 66.99)	54.78 (45.84, 64.94)	< 0.001
Triglyceride (mg/dl)	129.00 (91.00, 189.00)	130.00 (92.00, 186.00)	128.00 (89.00, 192.00)	0.66
Total cholesterol (mg/dl)	195.00 (167.00, 224.00)	206.00 (180.00, 233.00)	182.00 (155.00, 210.00)	< 0.0001
LDL (mg/dl)	111.00 (87.00, 137.00)	115.00 (92.00, 142.00)	106.00 (81.00, 130.00)	< 0.0001
HDL (mg/dl)	53.00 (43.00, 65.00)	58.00 (48.00, 71.00)	46.00 (39.00, 56.00)	< 0.0001
Race/ethnicity	−	−	−	0.01
Non-Hispanic white	81.83 (75.76, 87.90)	54.83 (53.84, 55.81)	45.17 (44.19, 46.16)	−
Non-Hispanic black	7.71 (6.91, 8.52)	59.33 (57.62, 61.05)	40.67 (38.95, 42.38)	−
Mexican American	3.38 (2.75, 4.01)	55.20 (52.92, 57.48)	44.80 (42.52, 47.08)	−
Other Race	7.07 (6.25, 7.90)	57.21 (53.87, 60.56)	42.79 (39.44, 46.13)	−
Marital status	−	−	−	< 0.001
Cohabited	1.83 (1.39, 2.26)	37.39 (28.97, 45.81)	62.61 (54.19, 71.03)	−
Divorced	11.43 (10.46, 12.39)	64.40 (61.22, 67.58)	35.60 (32.42, 38.78)	−
Married	62.06 (57.78, 66.33)	45.53 (44.59, 46.46)	54.47 (53.54, 55.41)	−
Separated	1.25 (1.06, 1.45)	57.09 (48.40, 65.77)	42.91 (34.23, 51.60)	−
Single	3.06 (2.68, 3.43)	54.07 (47.34, 60.81)	45.93 (39.19, 52.66)	−
Widowed	20.38 (18.94, 21.83)	81.91 (80.41, 83.40)	18.09 (16.60, 19.59)	−
Income	−	−	−	< 0.001
0–19,999	19.76 (17.94, 21.58)	66.54 (64.75, 68.33)	33.46 (31.67, 35.25)	−
20,000–$44,999	36.04 (33.48, 38.61)	57.59 (56.24, 58.94)	42.41 (41.06, 43.76)	−
45,000–74,999	20.55 (18.68, 22.42)	49.57 (47.65, 51.49)	50.43 (48.51, 52.35)	−
=$75,000	23.65 (21.39, 25.90)	47.63 (45.80, 49.45)	52.37 (50.55, 54.20)	−
Smoke	−	−	−	< 0.001
Never	48.17 (45.39, 50.95)	68.21 (66.85, 69.57)	31.79 (30.43, 33.15)	−
Former	41.22 (38.38, 44.05)	42.25 (40.41, 44.09)	57.75 (55.91, 59.59)	−
Now	10.56 (9.63, 11.49)	47.81 (44.25, 51.38)	52.19 (48.62, 55.75)	−

LDL, low density lipoprotein; HDL, high-density lipoprotein, GNRI, geriatric nutritional risk index; DII, dietary inflammatory index; HEI, healthy eating index; BMI, body mass index.

*P-values were calculated by Rao-Scott chi-square test and Wilcoxon rank-sum test.

**TABLE 2:** Baseline disease characteristics of all participants in this study.

Characteristics	Total	Female	Male	*P*-value*
Hyperlipidemia	−	−	−	<0.001
No	16.09 (14.81, 17.37)	47.89 (45.52, 50.26)	52.11 (49.74, 54.48)	−
Yes	83.91 (79.08, 88.74)	56.79 (55.81, 57.76)	43.21 (42.24, 44.19)	−
Hypertension	−	−	−	<0.001
No	29.79 (27.52, 32.07)	51.30 (49.47, 53.13)	48.70 (46.87, 50.53)	−
Yes	70.21 (66.23, 74.18)	57.08 (55.99, 58.16)	42.92 (41.84, 44.01)	−
DM	−	−	−	<0.001
No	63.35 (59.48, 67.21)	58.00 (56.83, 59.18)	42.00 (40.82, 43.17)	−
DM	26.27 (24.51, 28.04)	50.11 (47.79, 52.43)	49.89 (47.57, 52.21)	−
IFG	6.56 (5.72, 7.40)	48.80 (43.52, 54.09)	51.20 (45.91, 56.48)	−
IGT	3.82 (3.20, 4.44)	58.74 (54.20, 63.27)	41.26 (36.73, 45.80)	−
Cause of death	−	−	−	<0.001
Cancer	6.99 (6.30, 7.68)	42.56 (38.50, 46.62)	57.44 (53.38, 61.50)	−
CCVD	10.47 (9.51, 11.43)	50.44 (47.61, 53.27)	49.56 (46.73, 52.39)	−
NO	68.06 (63.73, 72.39)	57.22 (56.16, 58.27)	42.78 (41.73, 43.84)	−
Other	14.48 (13.31, 15.64)	56.34 (53.84, 58.85)	43.66 (41.15, 46.16)	−

DM, diabetes mellitus; IFG, impaired fasting glycemia; IGT, impaired glucose tolerance; CCVD, cardiovascular and cerebrovascular diseases.

*P-values were calculated by Rao-Scott chi-square test and Wilcoxon rank-sum test.

**FIGURE 2:**
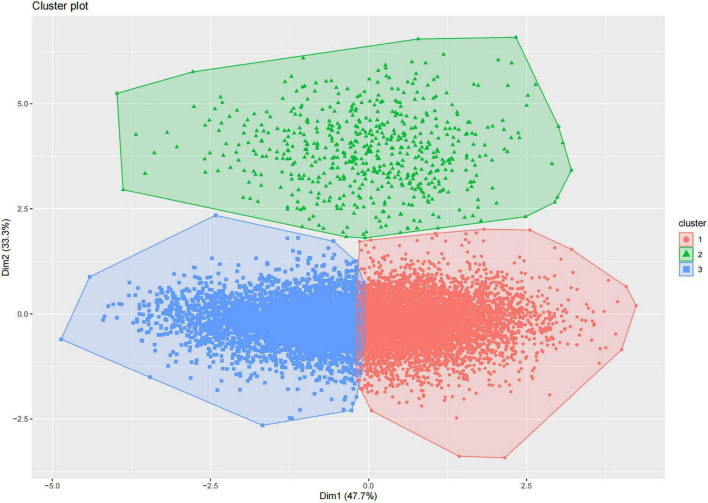
K-means clustering of participants based on three nutrition- and diet-related scores, and visualization. Red dots indicate cluster 1 [higher healthy eating index (HEI), lower dietary inflammatory index (DII)]; green dots indicate cluster 2 [lower geriatric nutritional risk index (GNRI)]; and blue dots indicate cluster 3 (lower HEI, higher DII).

We classified the 12,724 participants into three clusters using k-means clustering. The graph of the classification results is shown in Figure 2.

**FIGURE 3:**
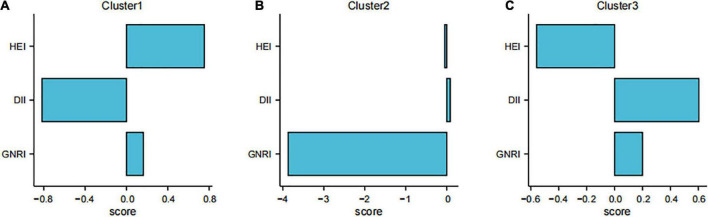
Distribution of cluster centers for the three scores in different clusters. **(A)** Cluster 1 [higher healthy eating index (HEI), lower dietary inflammatory index (DII)]; **(B)** Cluster 2 [lower geriatric nutritional risk index (GNRI)]; **(C)** Cluster 3 (lower HEI, higher DII).

The results of the analysis of the nutrition and diet-related index centers are shown in Figure 3 and Supplementary Table 2.

We categorized participants with lower DII and higher HEI as the first cluster, those with lower GNRI as the second cluster, and those with lower HEI and higher DII as the third cluster. Supplementary Table 3 shows the individual standardized scores. Table 3 shows the baseline characteristics of the study participants in the three weighted collections.

**TABLE 3:** Baseline characteristics of participants in the national health and nutrition examination survey (NHANES) stratified by the three clusters of nutrition- and diet-related scores.

Characteristics	Total	Cluster 1	Cluster 2	Cluster 3	*P*-value*
Age (years)	−	−	−	−	0.02
60–70	53.88 (50.49, 57.27)	54.73 (52.73, 56.73)	45.08 (38.90, 51.26)	53.83 (52.19, 55.46)	−
71–80	41.35 (38.87, 43.83)	40.58 (38.71, 42.46)	48.05 (42.27, 53.83)	41.49 (39.87, 43.10)	−
>80	4.77 (4.03, 5.52)	4.69 (3.78, 5.59)	6.87 (4.43, 9.32)	4.69 (4.02, 5.36)	−
BMI (kg/m2)	−	−	−	−	<0.001
<18.5	1.11 (0.91, 1.32)	1.15 (0.82, 1.48)	2.13 (0.93, 3.34)	1.01 (0.74, 1.28)	−
18.5–23.9	18.30 (16.84, 19.76)	20.15 (18.52, 21.79)	20.98 (16.47, 25.49)	16.60 (15.23, 17.97)	−
24–28	29.83 (27.80, 31.86)	32.40 (30.72, 34.08)	31.19 (25.93, 36.46)	27.63 (25.89, 29.37)	−
=28	50.76 (47.48, 54.03)	46.30 (44.58, 48.02)	45.70 (40.48, 50.91)	54.76 (52.92, 56.60)	−
Race/ethnicity	−	−	−	−	<0.001
Non-Hispanic white	81.83 (75.76, 87.90)	83.67 (82.00, 85.35)	70.72 (66.06, 75.39)	81.13 (79.12, 83.14)	−
Non-Hispanic black	7.71 (6.91, 8.52)	5.51 (4.74, 6.28)	16.77 (13.48, 20.05)	8.86 (7.63, 10.08)	−
Mexican American	3.38 (2.75, 4.01)	3.26 (2.62, 3.90)	3.05 (2.01, 4.08)	3.50 (2.67, 4.33)	−
Other Race	7.07 (6.25, 7.90)	7.55 (6.43, 8.68)	9.46 (6.23, 12.69)	6.51 (5.53, 7.49)	−
Marital status	−	−	−	−	<0.001
Cohabited	1.83 (1.39, 2.26)	1.48 (0.98, 1.99)	1.67 (0.34, 3.01)	2.12 (1.55, 2.68)	−
Divorced	11.43 (10.46, 12.39)	10.08 (8.85, 11.30)	14.36 (9.89, 18.82)	12.32 (11.15, 13.49)	−
Married	62.06 (57.78, 66.33)	65.83 (63.60, 68.05)	53.14 (47.39, 58.89)	59.62 (57.95, 61.30)	−
Separated	1.25 (1.06, 1.45)	1.11 (0.78, 1.44)	2.73 (1.11, 4.36)	1.27 (1.04, 1.49)	−
Single	3.06 (2.68, 3.43)	2.91 (2.32, 3.50)	2.14 (1.22, 3.06)	3.24 (2.71, 3.77)	−
Widowed	20.38 (18.94, 21.83)	18.59 (16.99, 20.20)	25.96 (21.25, 30.67)	21.44 (20.11, 22.77)	−
Income	−	−	−	−	<0.001
0–19,999	19.76 (17.94, 21.58)	15.18 (13.59, 16.76)	26.57 (21.84, 31.30)	23.01 (21.33, 24.69)	−
20,000–$44,999	36.04 (33.48, 38.61)	33.21 (31.08, 35.33)	36.80 (31.12, 42.48)	38.30 (36.36, 40.24)	−
45,000–74,999	20.55 (18.68, 22.42)	20.99 (19.05, 22.93)	20.25 (14.99, 25.50)	20.21 (18.74, 21.69)	−
=$75,000	23.65 (21.39, 25.90)	30.62 (28.02, 33.22)	16.39 (12.30, 20.47)	18.48 (16.65, 20.31)	−
Hyperlipidemia	−	−	−	−	<0.001
No	16.09 (14.81, 17.37)	16.38 (14.96, 17.80)	54.26 (48.87, 59.64)	13.09 (12.11, 14.08)	
Yes	83.91 (79.08, 88.74)	83.62 (82.20, 85.04)	45.74 (40.36, 51.13)	86.91 (85.92, 87.89)	
Hypertension	−	−	−	−	<0.001
No	29.79 (27.52, 32.07)	32.33 (30.36, 34.30)	23.83 (18.15, 29.51)	28.16 (26.61, 29.70)	−
Yes	70.21 (66.23, 74.18)	67.67 (65.70, 69.64)	76.17 (70.49, 81.85)	71.84 (70.30, 73.39)	−
DM	−	−	−	−	<0.001
No	63.35 (59.48, 67.21)	65.15 (63.23, 67.07)	72.86 (67.89, 77.84)	61.18 (59.62, 62.75)	−
DM	26.27 (24.51, 28.04)	23.96 (22.44, 25.49)	26.47 (21.61, 31.33)	28.14 (26.75, 29.54)	−
IFG	6.56 (5.72, 7.40)	6.50 (5.55, 7.46)	0.08 (-0.05, 0.21)	7.08 (6.06, 8.09)	−
IGT	3.82 (3.20, 4.44)	4.38 (3.45, 5.31)	0.59 (0.01, 1.17)	3.60 (2.91, 4.28)	−
Cause of death	−	−	−	−	<0.001
Cancer	6.99 (6.30, 7.68)	5.95 (5.12, 6.78)	15.57 (11.86, 19.28)	7.22 (6.39, 8.05)	−
CCVD	10.47 (9.51, 11.43)	9.33 (8.31, 10.36)	10.41 (7.58, 13.25)	11.40 (10.36, 12o9.44)	−
NO	68.06 (63.73, 72.39)	72.84 (70.93, 74.76)	59.17 (53.64, 64.70)	64.80 (62.80, 66.81)	−
Other	14.48 (13.31, 15.64)	11.87 (10.72, 13.02)	14.85 (11.05, 18.65)	16.57 (15.39, 17.76)	−

GNRI, geriatric nutritional risk index; DII, dietary inflammatory index; HEI, healthy eating index; BMI, body mass index; DM, diabetes mellitus; IFG, impaired fasting glycemia; IGT, impaired glucose tolerance; CCVD, cardiovascular and cerebrovascular diseases.

*P-values were calculated by Rao-Scott chi-square test.

Compared to the other two clusters, the second cluster had a larger proportion of participants aged 71–80 years (48%), a smaller proportion of caucasians (70.72%), a smaller proportion of people with hyperlipidemia (45.7%), and a higher proportion of people with hypertension (76.1%). Caucasians comprised the majority of participants.

### Nutrition and diet indices related to mortality risk

**FIGURE 4:**
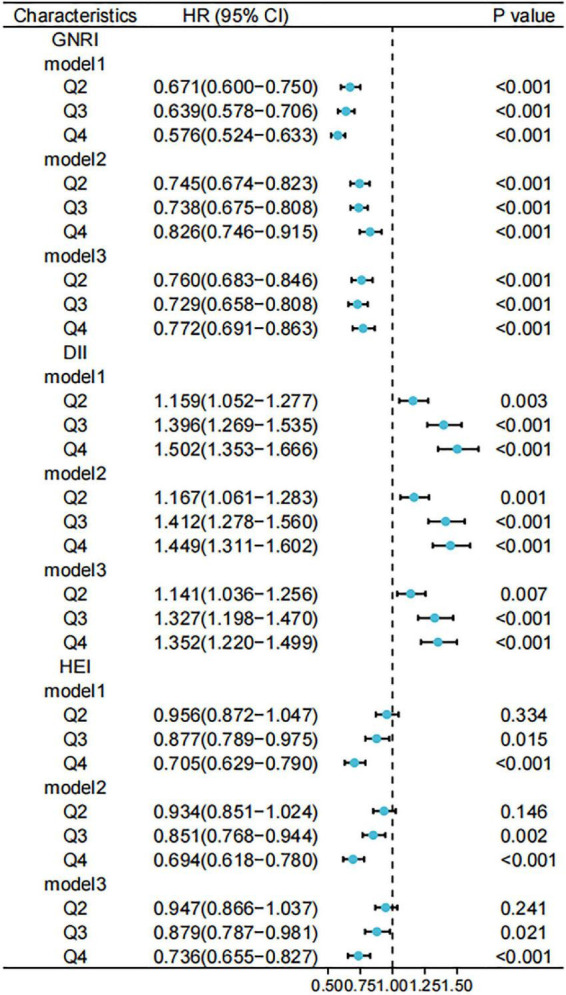
Relationship between different scores and risk of all-cause mortality. Standardized scores were divided into four groups (Q1, Q2, Q3, and Q4) according to the 25th, 50th, and 75th quartiles, with the Q1 group used as a reference. Model 1 was unadjusted for variables and Model 2 was adjusted for age, sex, race, marital status, and household income at baseline; Model 3 was further adjusted for history of hypertension, hyperlipidemia, and diabetes. GNRI, geriatric nutritional risk index; DII, dietary inflammatory index; HEI, healthy eating index.

Figure 4 shows forest plots of the three scores against all-cause mortality, considering the complex survey design.

**FIGURE 5:**
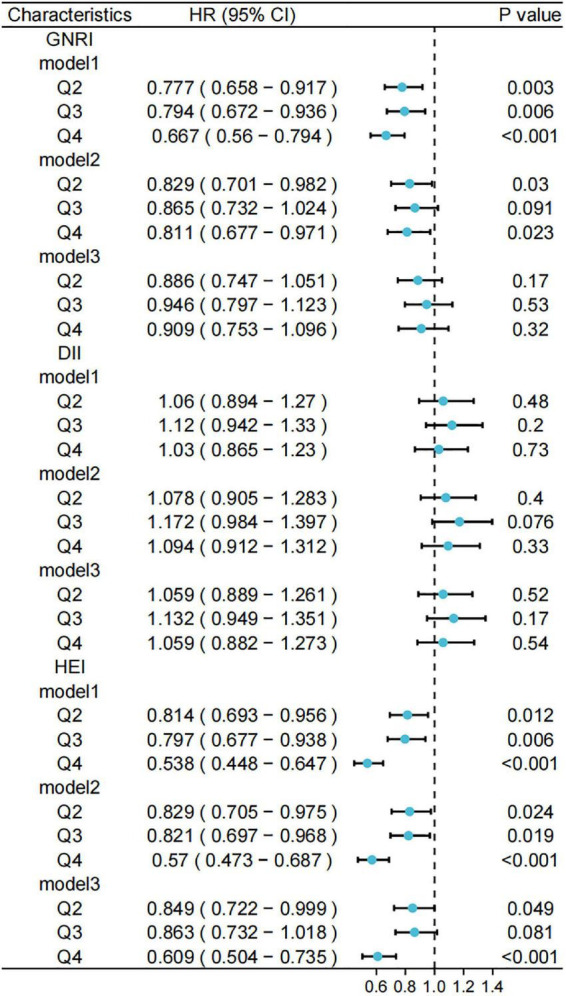
Relationship between different scores and risk of cancer mortality. Standardized scores were divided into four groups (Q1, Q2, Q3, and Q4) according to the 25th, 50th, and 75th quartiles, with the Q1 group used as a reference. Model 1 was unadjusted for variables and Model 2 was adjusted for age, sex, race, marital status, and household income at baseline; Model 3 was further adjusted for history of hypertension, hyperlipidemia, and diabetes. GNRI, geriatric nutritional risk index; DII, dietary inflammatory index; HEI, healthy eating index.

**FIGURE 6:**
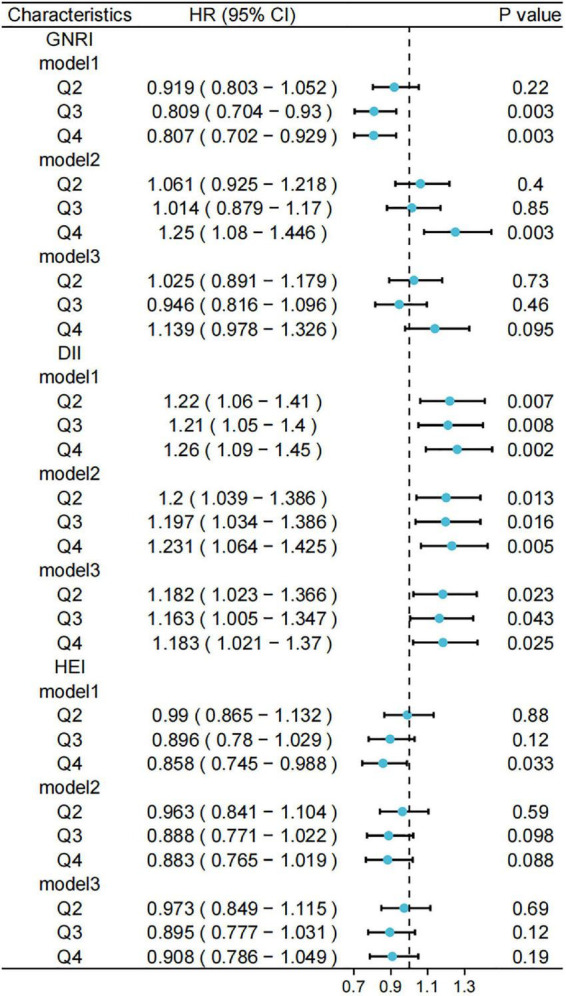
Relationship between different scores and risk of cardiovascular and cerebrovascular diseases (CCVD) mortality. Standardized scores were divided into four groups (Q1, Q2, Q3, and Q4) according to the 25th, 50th, and 75th quartiles, with the Q1 group used as a reference. Model 1 was unadjusted for variables and Model 2 was adjusted for age, sex, race, marital status, and household income at baseline; Model 3 was further adjusted for history of hypertension, hyperlipidemia, and diabetes. GNRI, geriatric nutritional risk index; DII, dietary inflammatory index; HEI, healthy eating index.

Furthermore, we used weighted to Cox regression calculate the HRs and 95% CIs. Figures 5, 6 show forest plots of the three scores (quartiles) against the risks of cancer-related and CCVD-related mortality.

Our unweighted competing risks model calculated the relationship between them.

We adjusted for participant baseline characteristics (sex and age) and patient-relevant medical history (hypertension and diabetes) to obtain HRs and 95% CIs for the different variable-adjusted indices versus all-cause mortality (Figure 4). We observed lower risks of death for participants with GNRI values in the second, third, and fourth quartiles compared to that in the lowest quartile in the model. This trend was observed in the entire model. For the DII, the risk of death was higher in the other quartiles than in the lowest quartile. For HEI, the risks of death were lower in the third (HR: 0.879, 95% CI: 0.787–0.981) and fourth quartiles (HR: 0.736, 95% CI: 0.655–0.827) compared to that in the lowest quartile.

Using competing risk models, we obtained forest plots of the different indices for cancer-related mortality risk (Figure 5). A higher GNRI was associated with a lower risk of cancer-related death only in the unadjusted model (HR: 0.667, 95% CI: 0.56–0.794), a trend that was not significant in the adjusted model. No models showed a significant association between the DII and the risk of cancer-related deaths. The risk of cancer death was lower in the highest quartile of HEI compared to that in the lowest quartile (HR: 0.609, 95% CI: 0.504–0.735). In contrast, the tendency for a reduced risk of cancer death in the second and third quartiles of HEI was more significant in models 1 and 2.

Figure 6 shows a forest plot of the risks of death associated with cardiovascular disease obtained from the unweighted competing risk model. After controlling for participant baseline information and chronic medical history, participants in the other three quartiles of the DII had an increased risk of death from cardiovascular disease compared to those in the lowest quartile (model 3, Q4; HR: 1.183, 95% CI: 1.021–1.37). For the GNRI and HEI, no significant trends were observed in the risk of death from cardiovascular disease in the adjusted models.

**FIGURE 7:**
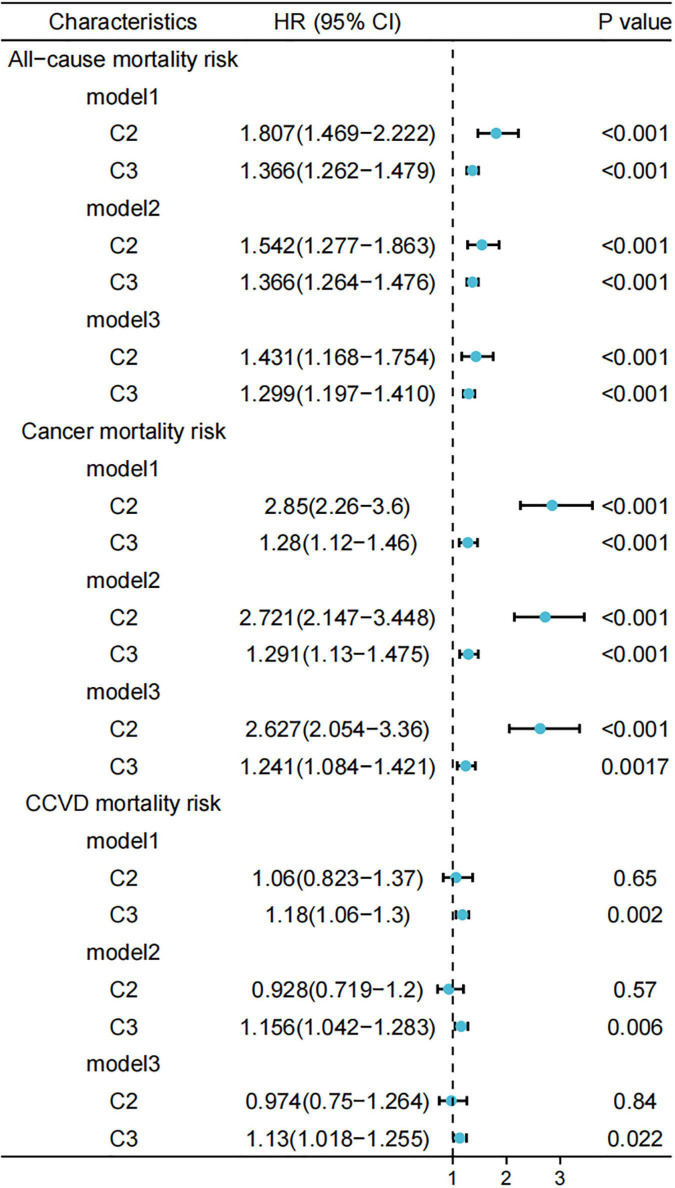
Relationship between different scores and risk of all-cause/cancer/cardiovascular and cerebrovascular diseases (CCVD) mortality. Standardized scores were divided into four groups (Q1, Q2, Q3, and Q4) according to the 25th, 50th, and 75th quartiles, with the Q1 group used as a reference. Model 1 was unadjusted for variables and Model 2 was adjusted for age, sex, race, marital status, and household income at baseline; Model 3 was further adjusted for history of hypertension, hyperlipidemia, and diabetes. GNRI, geriatric nutritional risk index; DII, dietary inflammatory index; HEI, healthy eating index.

After adjusting for multiple variables, we determined the relationships between different clusters and the risk of all-cause mortality using a weighted Cox proportional risk model. An unweighted competing risk model was used to assess the relationship between different sets and the risk of death from cancer and cardiovascular disease (Figure 7).

Compared to Cluster 1, the risk of all-cause mortality and cancer-related mortality was higher in Clusters 2 and 3 in the full model, with a more pronounced trend observed in Cluster 2 (model 3, HR: 2.627, 95% CI: 2.054–3.36). The risk of CCVD death was higher in Cluster 3 (model 3, HR: 1.13, 95% CI: 1.018–1.255) compared to Cluster 1, a trend that was not observed in Cluster 2.

## Discussion

This study assessed the relationship of nutritional status and dietary patterns with all-cause and specific causes of death in individuals aged >60 years. We considered the effects of combinations of dietary habits and nutritional status with different distributions in the population on the risk of death. Compared to those in the lowest quartile, the GNRI and HEI had a better prognosis in the higher levels. A higher DII score was associated with a higher risk of mortality. After unsupervised clustering of the population based on the three indicators (GNRI, DII, and HEI), the population was divided into three clusters. We found that Cluster 2, with a lower GNRI, was associated with higher all-cause and cancer risks of death compared to Cluster 1. This tendency was more pronounced than that in Cluster 3 (lower HEI, higher DII, and moderate GNRI).

The result of our study revealed a negative correlation between the GNRI and all-cause mortality. The GNRI was calculated based on the serum albumin concentration, height, and weight. The simplicity of this calculation has led to the widespread use of this nutritional indicator. Some studies have reported lower activity levels in older patients in the low GNRI group with heart failure (18). A low preoperative GNRI was associated with increased postoperative complications and poor prognosis (6, 19), which is consistent with our findings. It was also an independent prognostic factor for some cancers (20, 21). These are probably related to the anti-inflammatory and antioxidant physiological properties of serum albumin (22).

Dietary inflammatory index was positively associated with all-cause mortality in participants aged >60 years in the present study. A previous meta-analysis demonstrated that a pro-inflammatory diet was associated with increased risks of cardiovascular disease and mortality (23). Deng et al. also reported this correlation in the NHANES III database (24). This is consistent with our findings. We validated this finding using the latest mortality data from the NHANES database (follow-up to 2018). Pro-inflammatory diets were scored based on inflammatory factors, and their close relationship may explain the correlation between DII and mortality. For example, TNF-α and IL-1β play roles in vascular inflammation (25).

High-quality diets with higher HEI reduce all-cause mortality and the risk of cardiovascular disease and cancer death (26). A decreased HEI score indicates increased sugar or fat supply (27), leading to an increased risk of disease (28).

Owing to the individual variations in nutritional intakes and dietary patterns, the development of uniform standards provides a basis for studying the effects of diet on health (e.g., GNRI, DII, and HEI). However, single measures may affect the validity of the evaluation because they are developed for different populations or do not cover the full range of components. We clustered three different combinations of diet and nutrition scores om this study to evaluate the relationship between diet, nutrition, and health in older adults in a larger dimension. First, participants with a higher HEI and lower DII in Cluster 1 (moderate GNRI levels) had the best prognosis. Participants in Cluster 3 (higher DII, lower HEI, and average GNRI levels) had higher risks of all-cause mortality, cancer death, and CCVD death compared to that in Cluster 1.

In contrast, Cluster 2, with low GNRI (moderate DII and HEI), had a two-fold increased risk of cancer compared to Cluster 1. The high risk of CCVD death in cluster 3 may be associated with a high pro-inflammatory diet of inflammatory factors and increased energy supply from sugar or fat. These findings suggest that imbalances in nutritional status can have a significant impact on health.

This study had several strengths. First, the data were obtained from the newly updated NHANES database of survival data. Second, we classified the populations using unsupervised machine learning to identify distinctive characteristics. Additionally, we adjusted for the effects of covariates on the studied variables. However, this study has some additional limitations. First, the serum albumin measurements in the GNRI and diet collection in the diet model were not representative of the continuous state of the participants. Second, the k-means clustering method is sensitive to outliers; however, we normalized the data to reduce this possibility.

## Conclusion

This study explored the relationship between nutritional status and dietary patterns and the risk of all-cause and cause-specific mortality in people aged >60 years in the US. Healthy diet with high HEI and low DII are beneficial to health, whereas nutritional status requires modest maintenance. Our findings provide a new perspective to exploring the relationship between nutritional status, dietary patterns, and health.

## Data availability statement

Publicly available datasets were analyzed in this study. This data can be found here: https://www.cdc.gov/nchs/nhanes/.

## Ethics statement

The studies involving human participants were reviewed and approved by National Center for Health Statistics Research Ethics Review Board. National Health and Nutrition Examination Survey (NHANES) website (https://www.cdc.gov/nchs/nhanes/). The patients/participants provided their written informed consent to participate in this study.

## Author contributions

ZL and DX: conceptualization. ZL: methodology, resources, and visualization. W-XX: software. Y-JF, D-DW, and W-XX: validation. FD: investigation and supervision. DX: data curation. ZL and Y-JF: writing—original draft preparation. D-DW and FD: writing—review and editing. J-HT: project administration and funding acquisition. All authors have read and agreed to the published version of the manuscript.
