# Supplementary material for: Dietary patterns, nutritional status, and mortality risks among the elderly
Source: Front Nutr. 2022 Dec 9;9:963060. doi: 10.3389/fnut.2022.963060 (PMC9780274; doi:10.3389/fnut.2022.963060)
Supplement: Supplementary file 1 [file Table_1.DOCX]

Web sites for detailed nutritional status and dietary patterns from the NHANES survey in this study.

| Indicators | URLs |
| --- | --- |
| Albumin (g/l) | https://wwwn.cdc.gov/Nchs/Nhanes/1999-2000/LAB18.htm#LBDSALSI  https://wwwn.cdc.gov/Nchs/Nhanes/2001-2002/L40_B.htm#LBDSALSI  https://wwwn.cdc.gov/Nchs/Nhanes/2003-2004/L40_C.htm#LBDSALSI  https://wwwn.cdc.gov/Nchs/Nhanes/2005-2006/BIOPRO_D.htm#LBDSALSI  https://wwwn.cdc.gov/Nchs/Nhanes/2007-2008/BIOPRO_E.htm#LBDSALSI  https://wwwn.cdc.gov/Nchs/Nhanes/2009-2010/BIOPRO_F.htm#LBDSALSI  https://wwwn.cdc.gov/Nchs/Nhanes/2011-2012/BIOPRO_G.htm#LBDSALSI  https://wwwn.cdc.gov/Nchs/Nhanes/2013-2014/BIOPRO_H.htm#LBDSALSI  https://wwwn.cdc.gov/Nchs/Nhanes/2015-2016/BIOPRO_I.htm#LBDSALSI  https://wwwn.cdc.gov/Nchs/Nhanes/2017-2018/BIOPRO_J.htm#LBDSALSI |
| Height(cm) | https://wwwn.cdc.gov/Nchs/Nhanes/1999-2000/BMX.htm#BMIHT  https://wwwn.cdc.gov/Nchs/Nhanes/2001-2002/BMX_B.htm#BMIHT  https://wwwn.cdc.gov/Nchs/Nhanes/2003-2004/BMX_C.htm#BMIHT  https://wwwn.cdc.gov/Nchs/Nhanes/2005-2006/BMX_D.htm#BMIHT  https://wwwn.cdc.gov/Nchs/Nhanes/2007-2008/BMX_E.htm#BMIHT  https://wwwn.cdc.gov/Nchs/Nhanes/2009-2010/BMX_F.htm#BMIHT  https://wwwn.cdc.gov/Nchs/Nhanes/2011-2012/BMX_G.htm#BMIHT  https://wwwn.cdc.gov/Nchs/Nhanes/2013-2014/BMX_H.htm#BMIHT  https://wwwn.cdc.gov/Nchs/Nhanes/2015-2016/BMX_I.htm#BMIHT  https://wwwn.cdc.gov/Nchs/Nhanes/2017-2018/BMX_J.htm#BMIHT |
| Diet components | https://wwwn.cdc.gov/Nchs/Nhanes/1999-2000/DRXIFF.htm#DRXITFAT  https://wwwn.cdc.gov/Nchs/Nhanes/2001-2002/DRXIFF_B.htm#DRXITFAT  https://wwwn.cdc.gov/Nchs/Nhanes/2003-2004/DR1IFF_C.htm#DR1ITFAT  https://wwwn.cdc.gov/Nchs/Nhanes/2005-2006/DR1IFF_D.htm#DR1ITFAT  https://wwwn.cdc.gov/Nchs/Nhanes/2007-2008/DR1IFF_E.htm#DR1ITFAT  https://wwwn.cdc.gov/Nchs/Nhanes/2009-2010/DR1IFF_F.htm#DR1ITFAT  https://wwwn.cdc.gov/Nchs/Nhanes/2011-2012/DR1IFF_G.htm#DR1ITFAT  https://wwwn.cdc.gov/Nchs/Nhanes/2013-2014/DR1IFF_H.htm#DR1ITFAT  https://wwwn.cdc.gov/Nchs/Nhanes/2015-2016/DR1IFF_I.htm#DR1ITFAT  https://wwwn.cdc.gov/Nchs/Nhanes/2017-2018/DR1IFF_J.htm#DR1ITFAT |

Web sites of detailed covariates data from the NHANES survey in this study.

| Covariates data | URLs |
| --- | --- |
| Demographics | https://wwwn.cdc.gov/Nchs/Nhanes/1999-2000/DEMO.htm#DMDCITZN  https://wwwn.cdc.gov/Nchs/Nhanes/2001-2002/DEMO_B.htm#DMDCITZN  https://wwwn.cdc.gov/Nchs/Nhanes/2003-2004/DEMO_C.htm#DMDCITZN  https://wwwn.cdc.gov/Nchs/Nhanes/2005-2006/DEMO_D.htm#DMDCITZN  https://wwwn.cdc.gov/Nchs/Nhanes/2007-2008/DEMO_E.htm#DMDCITZN  https://wwwn.cdc.gov/Nchs/Nhanes/2009-2010/DEMO_F.htm#DMDCITZN  https://wwwn.cdc.gov/Nchs/Nhanes/2011-2012/DEMO_G.htm#DMDCITZN  https://wwwn.cdc.gov/Nchs/Nhanes/2013-2014/DEMO_H.htm#DMDCITZN  https://wwwn.cdc.gov/Nchs/Nhanes/2015-2016/DEMO_I.htm#DMDCITZN  https://wwwn.cdc.gov/Nchs/Nhanes/2017-2018/DEMO_J.htm#DMDCITZN |
| BMI | https://wwwn.cdc.gov/Nchs/Nhanes/1999-2000/BMX.htm#BMIHT  https://wwwn.cdc.gov/Nchs/Nhanes/2001-2002/BMX_B.htm#BMIHT  https://wwwn.cdc.gov/Nchs/Nhanes/2003-2004/BMX_C.htm#BMIHT  https://wwwn.cdc.gov/Nchs/Nhanes/2005-2006/BMX_D.htm#BMIHT  https://wwwn.cdc.gov/Nchs/Nhanes/2007-2008/BMX_E.htm#BMIHT  https://wwwn.cdc.gov/Nchs/Nhanes/2009-2010/BMX_F.htm#BMIHT  https://wwwn.cdc.gov/Nchs/Nhanes/2011-2012/BMX_G.htm#BMIHT  https://wwwn.cdc.gov/Nchs/Nhanes/2013-2014/BMX_H.htm#BMIHT  https://wwwn.cdc.gov/Nchs/Nhanes/2015-2016/BMX_I.htm#BMIHT  https://wwwn.cdc.gov/Nchs/Nhanes/2017-2018/BMX_J.htm#BMIHT |
| Smoking | https://wwwn.cdc.gov/Nchs/Nhanes/1999-2000/SMQ.htm#SMD030  https://wwwn.cdc.gov/Nchs/Nhanes/2001-2002/SMQ_B.htm#SMD030  https://wwwn.cdc.gov/Nchs/Nhanes/2003-2004/SMQ_C.htm#SMD030  https://wwwn.cdc.gov/Nchs/Nhanes/2005-2006/SMQ_D.htm#SMD030  https://wwwn.cdc.gov/Nchs/Nhanes/2007-2008/SMQ_E.htm#SMD030  https://wwwn.cdc.gov/Nchs/Nhanes/2009-2010/SMQ_F.htm#SMD030  https://wwwn.cdc.gov/Nchs/Nhanes/2011-2012/SMQ_G.htm#SMD030  https://wwwn.cdc.gov/Nchs/Nhanes/2013-2014/SMQ_H.htm#SMD030  https://wwwn.cdc.gov/Nchs/Nhanes/2015-2016/SMQ_I.htm#SMD030  https://wwwn.cdc.gov/Nchs/Nhanes/2017-2018/SMQ_J.htm#SMD030 |
| Hypertension | https://wwwn.cdc.gov/Nchs/Nhanes/1999-2000/BPQ.htm#BPQ030  https://wwwn.cdc.gov/Nchs/Nhanes/2001-2002/BPQ_B.htm#BPQ030  https://wwwn.cdc.gov/Nchs/Nhanes/2003-2004/BPQ_C.htm#BPQ030  https://wwwn.cdc.gov/Nchs/Nhanes/2005-2006/BPQ_D.htm#BPQ030  https://wwwn.cdc.gov/Nchs/Nhanes/2007-2008/BPQ_E.htm#BPQ030  https://wwwn.cdc.gov/Nchs/Nhanes/2009-2010/BPQ_F.htm#BPQ030  https://wwwn.cdc.gov/Nchs/Nhanes/2011-2012/BPQ_G.htm#BPQ030  https://wwwn.cdc.gov/Nchs/Nhanes/2013-2014/BPQ_H.htm#BPQ030  https://wwwn.cdc.gov/Nchs/Nhanes/2015-2016/BPQ_I.htm#BPQ030  https://wwwn.cdc.gov/Nchs/Nhanes/2017-2018/BPQ_J.htm#BPQ030 |
| Diabetes | https://wwwn.cdc.gov/Nchs/Nhanes/1999-2000/DIQ.htm#DIQ010  https://wwwn.cdc.gov/Nchs/Nhanes/2001-2002/DIQ_B.htm#DIQ010  https://wwwn.cdc.gov/Nchs/Nhanes/2003-2004/DIQ_C.htm#DIQ010  https://wwwn.cdc.gov/Nchs/Nhanes/2005-2006/DIQ_D.htm#DIQ010  https://wwwn.cdc.gov/Nchs/Nhanes/2007-2008/DIQ_E.htm#DIQ010  https://wwwn.cdc.gov/Nchs/Nhanes/2009-2010/DIQ_F.htm#DIQ010  https://wwwn.cdc.gov/Nchs/Nhanes/2011-2012/DIQ_G.htm#DIQ010  https://wwwn.cdc.gov/Nchs/Nhanes/2013-2014/DIQ_H.htm#DIQ010  https://wwwn.cdc.gov/Nchs/Nhanes/2015-2016/DIQ_I.htm#DIQ010  https://wwwn.cdc.gov/Nchs/Nhanes/2017-2018/DIQ_J.htm#DIQ010 |
| CVD,cardiovascular disease, Cancer | https://wwwn.cdc.gov/Nchs/Nhanes/1999-2000/MCQ.htm#MCQ160C  https://wwwn.cdc.gov/Nchs/Nhanes/2001-2002/MCQ_B.htm#MCQ160C  https://wwwn.cdc.gov/Nchs/Nhanes/2003-2004/MCQ_C.htm#MCQ160C  https://wwwn.cdc.gov/Nchs/Nhanes/2005-2006/MCQ_D.htm#MCQ160C  https://wwwn.cdc.gov/Nchs/Nhanes/2007-2008/MCQ_E.htm#MCQ160C  https://wwwn.cdc.gov/Nchs/Nhanes/2009-2010/MCQ_F.htm#MCQ160C  https://wwwn.cdc.gov/Nchs/Nhanes/2011-2012/MCQ_G.htm#MCQ160C  https://wwwn.cdc.gov/Nchs/Nhanes/2013-2014/MCQ_H.htm#MCQ160C  https://wwwn.cdc.gov/Nchs/Nhanes/2015-2016/MCQ_I.htm#MCQ160C  https://wwwn.cdc.gov/Nchs/Nhanes/2017-2018/MCQ_J.htm#MCQ160C |
| Laboratory | https://wwwn.cdc.gov/Nchs/Nhanes/1999-2000/LAB18.htm#LBXSTR  https://wwwn.cdc.gov/Nchs/Nhanes/2001-2002/L40_B.htm#LBXSTR  https://wwwn.cdc.gov/Nchs/Nhanes/2003-2004/L40_C.htm#LBXSTR  https://wwwn.cdc.gov/Nchs/Nhanes/2005-2006/BIOPRO_D.htm#LBXSTR  https://wwwn.cdc.gov/Nchs/Nhanes/2007-2008/BIOPRO_E.htm#LBXSTR  https://wwwn.cdc.gov/Nchs/Nhanes/2009-2010/BIOPRO_F.htm#LBXSTR  https://wwwn.cdc.gov/Nchs/Nhanes/2011-2012/BIOPRO_G.htm#LBXSTR  https://wwwn.cdc.gov/Nchs/Nhanes/2013-2014/BIOPRO_H.htm#LBXSTR  https://wwwn.cdc.gov/Nchs/Nhanes/2015-2016/BIOPRO_I.htm#LBXSTR  https://wwwn.cdc.gov/Nchs/Nhanes/2017-2018/BIOPRO_J.htm#LBXSTR |

CVD, cardiovascular diseases; BMI, body mass index.

**Table S1. Baseline characteristics of all participants in this study.**

|  | **Female** | | **Male** | |
| --- | --- | --- | --- | --- |
|  | **[ALL]** | **N** | **[ALL]** | **N** |
|  | **N=6414** |  | **N=6310** |  |
| Age | 71.1 (7.04) | 6414 | 71.2 (6.89) | 6310 |
| BMI | 29.4 (6.63) | 6414 | 28.5 (5.32) | 6310 |
| GNRI | 114 (18.2) | 6414 | 113 (16.9) | 6310 |
| DII | 1.51 (1.86) | 6414 | 0.87 (1.95) | 6310 |
| HEI | 57.7 (13.3) | 6414 | 55.2 (13.2) | 6310 |
| Triglyceride (mean (SD)) | 152.92 (91.98) |  | 155.21 (116.05) |  |
| Total cholesterol (mean (SD)) | 206.73 (42.56) |  | 185.80 (41.83) |  |
| LDL(mean (SD)) | 118.10 (37.95) |  | 108.78 (37.08) |  |
| HDL(mean (SD)) | 59.39 (16.86) |  | 48.91 (14.49) |  |
| Eth: |  | 6414 |  | 6310 |
| black | 1189 (18.5%) |  | 1183 (18.7%) |  |
| mexican | 897 (14.0%) |  | 834 (13.2%) |  |
| other | 819 (12.8%) |  | 700 (11.1%) |  |
| white | 3509 (54.7%) |  | 3593 (56.9%) |  |
| Marital: |  | 6414 |  | 6310 |
| Cohabited | 84 (1.31%) |  | 158 (2.50%) |  |
| Divorced | 899 (14.0%) |  | 654 (10.4%) |  |
| Married | 2832 (44.2%) |  | 4452 (70.6%) |  |
| Separated | 164 (2.56%) |  | 122 (1.93%) |  |
| Single | 273 (4.26%) |  | 231 (3.66%) |  |
| Widowed | 2162 (33.7%) |  | 693 (11.0%) |  |
| Income: |  | 6414 |  | 6310 |
| $0-19,999 | 2038 (31.8%) |  | 1542 (24.4%) |  |
| $20,000-$44,999 | 2406 (37.5%) |  | 2400 (38.0%) |  |
| $45,000-74,999 | 1010 (15.7%) |  | 1206 (19.1%) |  |
| >=$75,000 | 960 (15.0%) |  | 1162 (18.4%) |  |
| Hyperlipidemia: |  | 6414 |  | 6310 |
| no | 943 (14.7%) |  | 1323 (21.0%) |  |
| yes | 5471 (85.3%) |  | 4987 (79.0%) |  |
| Hypertension: |  | 6414 |  | 6310 |
| no | 1535 (23.9%) |  | 1863 (29.5%) |  |
| yes | 4879 (76.1%) |  | 4447 (70.5%) |  |
| DM: |  | 6414 |  | 6310 |
| no | 3943 (61.5%) |  | 3613 (57.3%) |  |
| DM | 1888 (29.4%) |  | 2069 (32.8%) |  |
| IFG | 331 (5.16%) |  | 418 (6.62%) |  |
| IGT | 252 (3.93%) |  | 210 (3.33%) |  |
| Smoke: |  | 6411 |  | 6303 |
| never | 3938 (61.4%) |  | 2126 (33.7%) |  |
| former | 1876 (29.3%) |  | 3298 (52.3%) |  |
| now | 597 (9.31%) |  | 879 (13.9%) |  |
| Cause of Death: |  | 6414 |  | 6310 |
| cancer | 392 (6.11%) |  | 639 (10.1%) |  |
| CVD | 686 (10.7%) |  | 887 (14.1%) |  |
| no | 4307 (67.1%) |  | 3686 (58.4%) |  |
| other | 1029 (16.0%) |  | 1098 (17.4%) |  |

GNRI, Geriatric Nutritional Risk Index; DII, Dietary Inflammatory Index; HEI, Healthy Eating Index；BMI, body mass index; DM, diabetes mellitus; IFG,Impaired Fasting Glycaemia; IGT, Impaired Glucose Tolerance; CCVD, [cardiovascular](javascript:;) [and](javascript:;) [cerebrovascular](javascript:;) [diseases](javascript:;).

| Table S2. Centres of the three clusters of the three studied scores with prior log-transformation and standardization. | | | |
| --- | --- | --- | --- |
| Variables | Cluster 1 | Cluster 2 | Cluster 3 |
| GNRI | 0.16 | -3.87 | 0.20 |
| DII | -0.82 | 0.09 | 0.60 |
| HEI | 0.75 | -0.05 | -0.56 |
| GNRI,Geriatric Nutritional Risk Index; DII, Dietary Inflammatory Index; HEI, Healthy Eating Index. | | | |

| Table S3. The distribution of the standardization of the different scores studied. | | | | | |
| --- | --- | --- | --- | --- | --- |
| Variables | Min | 1^th^ Quantile | Median | 3^th^ Quantile | Max |
| GNRI | -6.524 | -0.184 | 0.139 | 0.466 | 3.477 |
| DII | -4.815 | -0.586 | 0.213 | 0.766 | 1.721 |
| HEI | -4.636 | -0.637 | 0.073 | 0.734 | 2.496 |
| GNRI,Geriatric Nutritional Risk Index; DII, Dietary Inflammatory Index; HEI, Healthy Eating Index. | | | | | |
